# Gender, reproductive output covariation and their role on gene diversity of *Pinus koraiensis* seed orchard crops

**DOI:** 10.1186/s12870-020-02632-9

**Published:** 2020-09-07

**Authors:** Ji-Min Park, Hye-In Kang, Da-Bin Yeom, Kyu-Suk Kang, Yousry A. El-Kassaby, Kyung-Mi Lee

**Affiliations:** 1 Department of Forest Sciences, Seoul National University, Gwanak-ro 1, Gwanak-gu, Seoul, 08826 Republic of Korea; 2 Department of Forest Genetic Resources, National Institute of Forest Science, Suwon, 16631 Republic of Korea; 3 Division of Plant Resources, Korean National Arboretum, Pocheon, 11186 Republic of Korea; 4 Department of Forest and Conservation Sciences, Faculty of Forestry, The University of British Columbia, Vancouver, BC V6T 1Z4 Canada

**Keywords:** Effective population size, Fertility variation, Flowering assessment, Reproductive success, Parental balance, Korean pine

## Abstract

**Background:**

Gender and fertility variation have an impact on mating dynamics in a population because they affect the gene exchange among parental members and the genetic composition of the resultant seed crops. Fertility is the proportional gametic contribution of parents to their progeny. An effective number of parents, derivative of effective population size, is the probability that two alleles randomly chosen from the gamete gene pool originated from the same parent. The effective number of parents is directly related to the fertility variation among parents, which should be monitored for manipulating gene diversity of seed crops. We formulated a fundamental equation of estimating the effective number of parents and applied it to a seed production population.

**Results:**

Effective number of parents (*N*_*p*_) was derived from fertility variation (Ψ) considering covariance (correlation coefficient, *r*) between maternal and paternal fertility. The Ψ was calculated from the coefficient of variation in reproductive outputs and divided into female (ψ_*f*_) and male (ψ_*m*_) fertility variation in the population under study. The *N*_*p*_ was estimated from the parental Ψ estimated by the fertility variation of maternal (ψ_*f*_) and paternal (ψ_*m*_) parents. The gene diversity of seed crops was monitored by Ψ and *N*_*p*_. in a 1.5 generation *Pinus koraiensis* seed orchard as a case of monoecious species. A large variation of female and male strobili production was observed among the studied 52 parents over four consecutive years, showing statistically significant differences across all studied years. Parental balance curve showed greater distortion in paternal than maternal parents. The Ψ ranged from 1.879 to 4.035 with greater ψ_*m*_ than ψ_*f*_, and the *N*_*p*_ varied from 14.8 to 36.8. When pooled, the relative effective number of parents was improved as 80.0% of the census number.

**Conclusions:**

We recommend the use of fertility variation (i.e., *CV*, Ψ), Person’s product-moment correlation (*r*), and effective number of parents (*N*_*p*_) as tools for gauging gene diversity of seed crops in production populations. For increasing *N*_*p*_ and gene diversity, additional management options such as mixing seed-lots, equal cone harvest and application of supplemental-mass-pollination are recommended.

## Background

Gender and reproductive output variation have a profound impact on the mating dynamics in a population, such as forest tree seed orchards, as they affect the gene exchange among the parental populations’ members and the genetic composition of the resultant seed crops [1–3]. In seed orchards, the theoretical expectation of reproductive output equality (uniform production of male and female gametes) is hardly fulfilled [4] and the extent of this variation has been the subject of extensive research [5–11]. Quantitative assessment of reproductive output in conifer seed orchards clearly indicated the presence of sexual asymmetry between female and male fertility [7, 8, 12, 13]; however, this asymmetry could be further separated if the observed reproductive output variation is either negatively or positively correlated (i.e., covariation).

Covariance is a measure of the joint variability of both variables (e.g., female and male fertility) in statistical probability theory. If greater values of female fertility correspond with greater values of male fertility, the covariance of female and male is positive. Conversely, when female and male fertilities tend to show opposite behavior, the covariance is negative. The sign of the covariance therefore shows the tendency in their linear relationship. The magnitude of the covariance is not easy to interpret because it is not normalized and hence depends on female and male fertilities magnitudes. However, correlation coefficient (i.e., the normalized version of covariance) shows the strength of the linear relation by its magnitude.

Effective population size (*Ne*) is one of the key genetic indicators in plant breeding and conservation programs, and it is central to population genetics and evolutionary biology [14, 15]. *Ne* quantifies the magnitude of genetic drift and inbreeding in the population under study. Several theoretical effective number extensions have been made such as inbreeding effective population size *Ne*^(*i*)^, variance effective population size *Ne*^(*v*)^ [16], selection effective population size [17], and status number [18]. In practice, *Ne* is, however, notoriously difficult to estimate. In forestry context, Kang [19] indicated that the effective number of parents is the number of individuals in which an idealized population would produce the same number of offspring (sibs) as the real population.

*Pinus koraiensis* Siebold & Zucc, commonly known as Korean pine, is a coniferous white-pine tree species native to the temperate rainforests of Korea, Japan, and the Ussuri River basin of China and Russia. Primordia differentiation starts in year-1, pollination and fertilization is completed in year-2, and seed and cone development is completed in year-3 [20]. The Korean pine occupies more than 25% of the total forest area in South Korea and is managed for timber and seed production for furniture, construction and human consumption [21–23]. In South Korea, Korean pine genetic improvement started with the selection of 300 phenotypically superior individuals forming the breeding population in 1959 (i.e., plus-trees) and the establishment of open-pollinated progeny tests in 1975 [24]. In 1970, the first-generation seed orchard was established by grafts of the selected plus trees. Volume growth, tree trunk volume, was the main selection criterion used for the transition from first- to 1.5-generation seed orchards [23, 24]. Thus, the 1.5-generation seed orchard represents the second-cycle of the program’s seed orchard and superior parents were selected based on their growth characteristics.

Investigating the extent of reproductive output (strobili and seed production) variation and covariation as well as the genetic composition of seed crops are essential to ensure the genetic quality of reforestation stock. However, the reproductive output and success information of *P. koraiensis* seed orchards have been limited. Here, we utilized a 1.5-generation *P. koraiensis* clonal seed orchard to develop a framework for estimating: 1) the effective number of parents (i.e., effective population size) considering the observed gender and reproductive output variation and covariation and 2) the gene diversity of the orchard’s seed crops. To do so, over four consecutive years, we surveyed strobili production difference and correlation of the seed orchard’s 52 parents (clones) and investigated gender (female and male strobili production) and reproductive out variation and covariation.

## Results

### Fertility covariation and effective number of parents

**Fig. 1:**
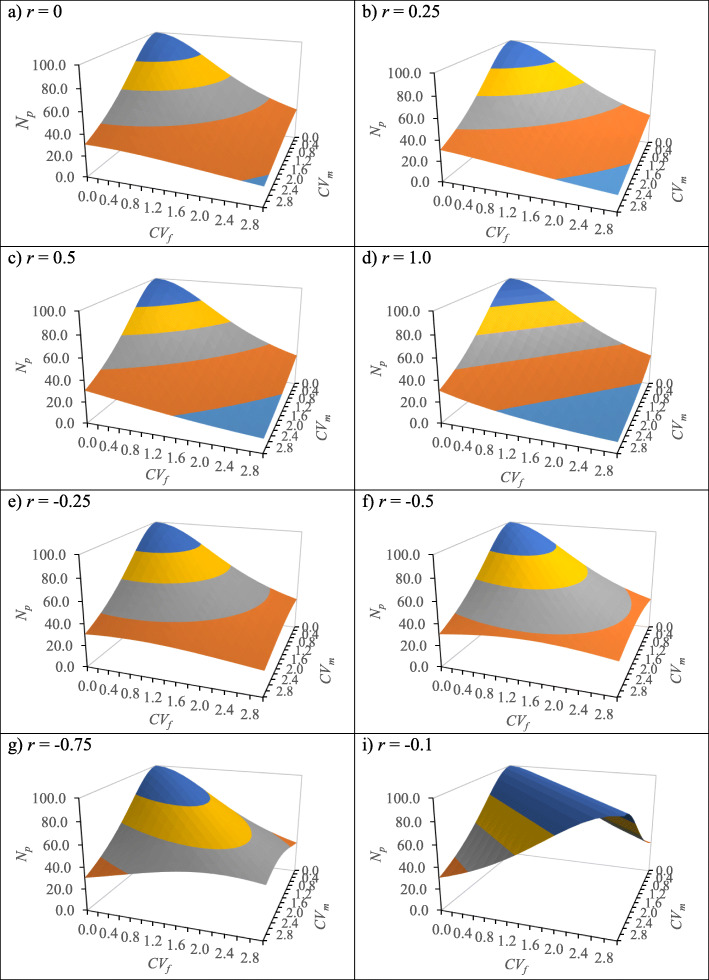
Stochastic simulation of the effective number of parents (*N*_*p*_) with female and male fertility variation (*CV*_*f*_, *CV*_*m*_) under various covariation (correlation coefficients, *r*) between female and male reproductive outputs. The census number was set to be 100 (*N* = 100) in the population

Under various scenarios of female and male fertility covariation (i.e., joint variability of female and male fertility related to correlation), the effective number of parents was stochastically simulated under a range of correlation coefficients (− 1.0 ≤ r ≤ 1.0) (Fig. 1). Generally, under no or limited female and male parents reproductive output fertility covariation, the effective number of parents (*N*_*p*_) was always equivalent to the census number (*N*) as the seed orchard parents are unrelated and assumed to be non-inbred (Fig. 1).

Positive female and male parents reproductive output fertility covariation increased the sibling coefficient (Ψ; parental fertility variation) as Ψ is affected by variation in both female (ψ_*f*_) and male (ψ_*m*_), causing the effective number of parents (*N*_*p*_) declined (Fig. 1a – 1.d), compared to equal fertility with no correlation. On the other hand, negative female and male parents reproductive output fertility covariation mitigated the asymmetrical variation between ψ_*f*_ and ψ_*m*_ (fertility variation imbalance), resulting the incremental increase of the effective number of parents (Fig. 1e – 1.i).

**Fig. 2:**
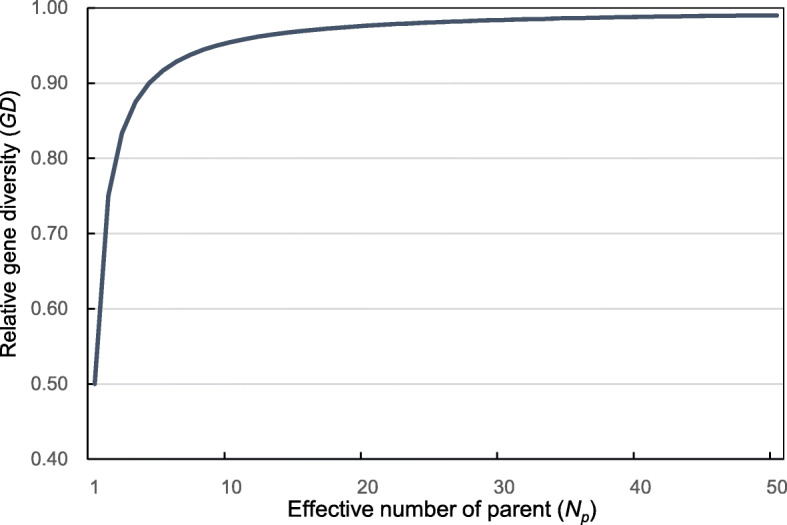
Relationship between seed orchard crops’ effective number of parents (*N*_*p*_) and gene diversity (*GD*) relative to their parental papulation

Knowledge regarding the extent of gene diversity loss (*GD*) when genes are transmitted from orchard parents to their progeny is valuable. The *GD* is estimated using Eq. (8) for new seed orchard establishment plans. If 5% loss of gene diversity is tolerable, then the effective number of parents *N*_*p*_ of 10 would be sufficient in providing the desired seed crop’s gene diversity (Fig. 2). However, striving to reach higher effective number of parents is preferable to ensure capturing reasonable level of gene diversity.

### Case study: *Pinus koraiensis* seed orchard

The average number of female strobili per ramet (a member of a clone) fluctuated across the studied years, with 2015 and 2016 representing the highest and lowest production with clone averages of 2.99 and 0.33, respectively (Table 1). The clonal average number of male strobili over years produced striking differences with 2017 and 2014, showing the highest and lowest production with averages of 1912.2 and 1.82, respectively (Table 1). The female and male strobili production over the studied years was low and negating panmixia expectations in the 1.5 generation clonal seed orchard of *P. koraiensis*. This was similar situation with previous observation in the first-generation clonal seed orchards of the same species.

**Table 1:** Average production of female and male strobili per ramet and correlation coefficient estimates (*r*) for four successive years in the 1.5-generation *P. koraiensis* clonal seed orchard

	2014	2015	2016	2017	Pooled
Female strobili	2.69	2.99	0.33	0.92	1.73
Male strobili	1.82	84.44	51.24	1912.18	512.44
*r*^a^	−0.02	0.36	−0.14	0.09	0.22

^a^Person’s correlation coefficient between female and male strobilus production

**Fig. 3:**
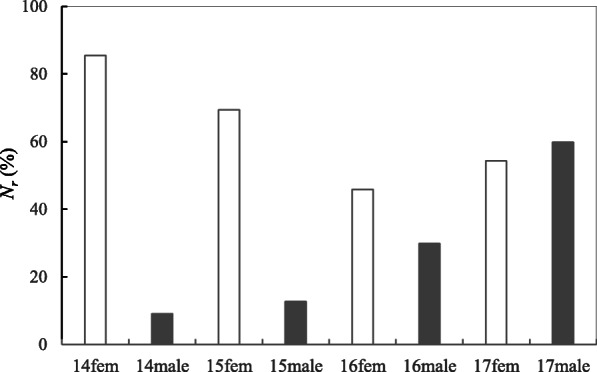
Relative effective number of parents (*N*_*r*_, relative to census number) for female and male parents in the 1.5-generation *P. koraiensis* clonal seed orchard

The effective number of female parents (*N*_*p*_^(*f*)^) was higher than that of male parents (*N*_*p*_^(*m*)^) except in the year 2017 (Table 2, Fig. 3). The relative effective number of female parents ranged 45.9% in 2016 (poor year) to 85.5% in 2014 (good year), and the expected loss of gene diversity (*GD*) for female and male parents were 1.1 and 1.6%, respectively, which was not so alarming for a 52 clonal seed orchard (Table 2). The clonal effective number of parents (*N*_*p*_) under female and male strobili production covariation varied between 14.8 and 36.8 for 2014 and 2017 across the four studied years (Table 3) where *N*_*p*_ was calculated using the *CV* and *r* of female and male strobili production (see Eq. 6). The seed crops’ loss of gene diversity (*GD*) varied between 3.4 and 1.4% for 2014 and 2017, presenting higher than expected values for female and male parents and indicating the effect of covariation (correlation) between female and male fertility.

**Table 2:** Coefficient of variation for female (*CV*_*f*_) and male (*CV*_*m*_) strobilus production, sibling coefficient of female (ψ_*f*_) and male (ψ_*m*_), effective number of female (*N*_*p*_^(*f*)^) and male (*N*_*p*_^(*m*)^) parents, relative effective number of female (*N*_*r*_^(*f*)^) and male (*N*_*r*_^(*m*)^) parents, and gene diversity (*GD*) in the 1.5-generation *P. koraiensis* clonal seed orchard (*N* = 52)

	2014		2015		2016		2017		Pooled	
	Female	Male	Female	Male	Female	Male	Female	Male	Female	Male
*CV*_*f*_ and *CV*_*m*_	0.412	3.158	0.664	2.620	1.087	1.534	0.918	0.820	0.403	0.831
ψ_*f*_ and ψ_*m*_	1.169	10.972	1.441	7.864	2.181	3.353	1.843	1.673	1.162	1.690
*N*_*p*_^(*f*)^ and *N*_*p*_^(*m*)^	44.5	4.7	36.1	6.6	23.8	15.5	28.2	31.1	44.7	30.8
*N*_*r*_^(*f*)^ and *N*_*r*_^(*m*)a^	85.5	9.1	69.4	12.7	45.9	29.8	54.2	59.8	86.0	59.2
*GD*	0.989	0.895	0.986	0.924	0.979	0.968	0.982	0.984	0.989	0.984

^a^*N*_*r*_^(*f*)^ and *N*_*r*_^(*m*)^ are relative percentages (%) to the census number (*N*)

**Table 3:** Clonal sibling coefficient (Ψ), parental effective number of parents (*N*_*p*_), relative effective number of parents (*N*_*r*_), and loss of gene diversity (*GD*) in the 1.5-generation *P. koraiensis* clonal seed orchard

	2014	2015	2016	2017	Pooled
Ψ	4.035	3.326	2.384	1.879	1.713
*N*_*p*_	14.8	16.6	29.4	36.8	41.6
*N*_*r*_ (%)	28.4	31.9	56.6	70.8	80.0
*GD* loss (%)	3.4	3.0	1.7	1.4	1.2

**Fig. 4:**
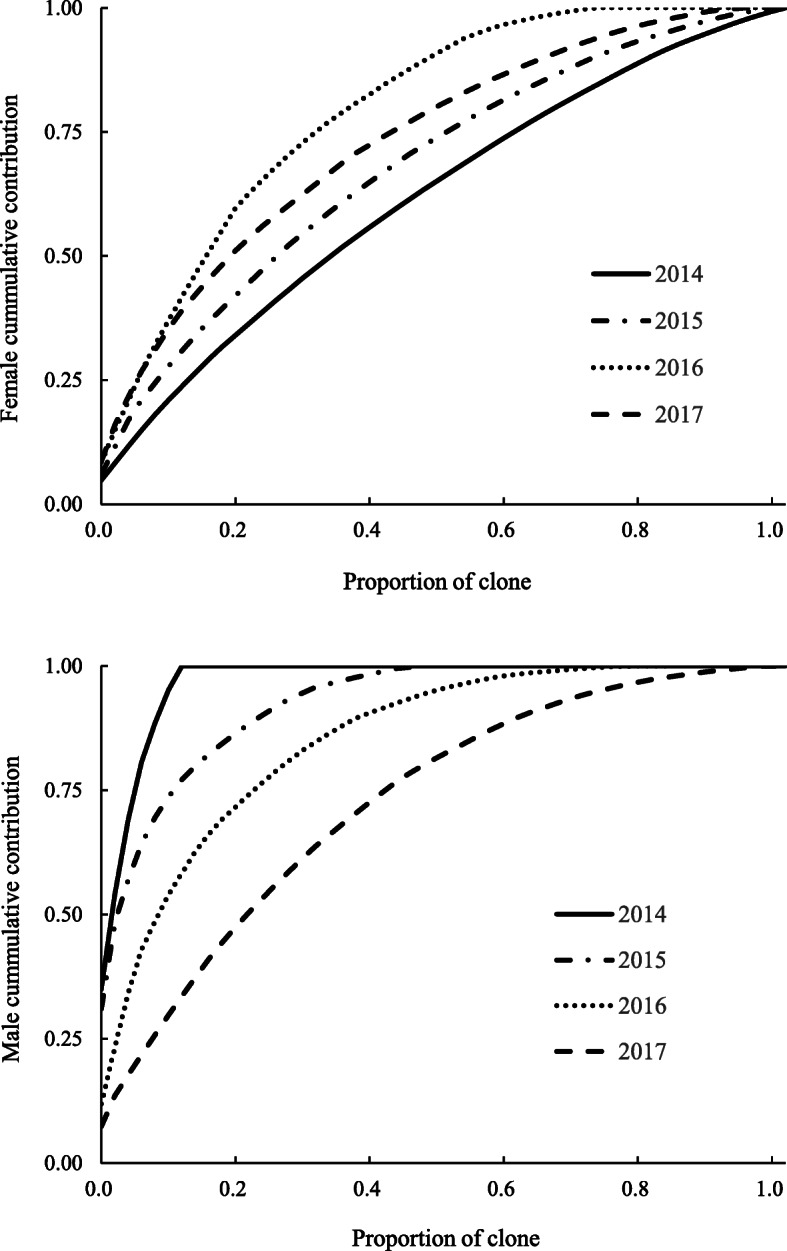
Cumulative contribution curves for female (upper) and male (lower) strobili production in the 1.5-generation *P. koraiensis* clonal seed orchard from 2014 to 2017

The parental balance curves showed that clonal cumulative gamete contribution was far from expectation (i.e., equal contribution) specifically for 2016 female and 2014 male (Fig. 4). The male strobili production cumulative curves showed greater distortion than that for female. The top 20% of clone contributed 59.6% of female strobili production (2016) while 86.4% of male production (2015). On the other hand, male strobili production was limited to extremely limited clones as only two clones contributed 50% of total production (Fig. 4).

Parental contribution as males, females or both sexes should influence the seed crop’s genetic composition, and this can be determined with assessment of the orchard’s initial reproduction and throughout the cone crop development. The current study indicated that there were 8 clones (15.4%) consistently ranked high on the gametic contribution. On the other hand, 8 clones were persistently ranked low across the orchard reproduction years, which could contribute to the needed reproductive output assessment. The genetic worth of orchards’ seed crops is a function of parental gametic contribution and their respective breeding value, thus sibling coefficient could be one of the criteria needed for evaluating the genetic composition as it determines parental gametic contribution [19]. Large variation among orchard parents’ gametic contribution is common and widely reported in many seed orchards [25]. Thus, an evaluation of seed crops’ genetic composition should consider the entire parental population as an analytical unit of gametic and genetic contribution.

**Fig. 5:**
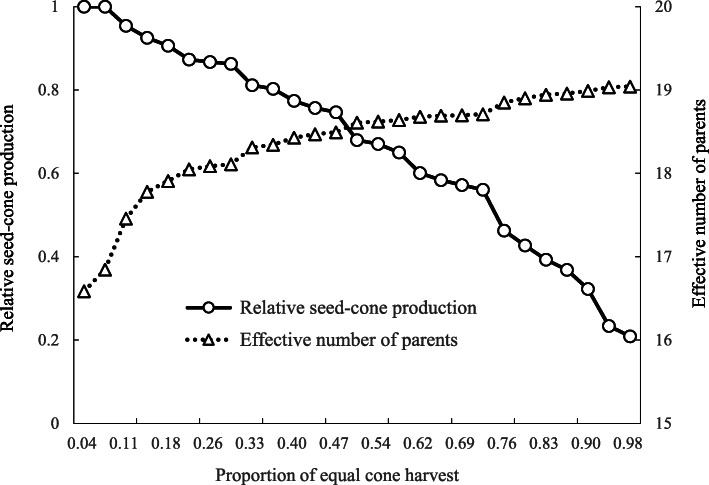
Trade-off between seed-cone production and effective number of parents by an equal seed-cone harvest exposed for a good crop year (2015) in the 1.5-generation seed orchard of *P. koraiensis*

By knowing the magnitude of fertility variation among individuals in a seed orchard, the census number to collect seed-cones could be chosen to achieve satisfactory gene diversity of seed crops [26]. We exposed the practice of equal seed-cone harvest for a good crop year (2015) in the *P. koraiensis* seed orchard. The equalizing of female fertility should be preferentially set to the most-fertile female parents, and the male fertilities were not changed. When the proportion of equal seed-cone harvest increased, the effective number of parents increased, but the relative seed-cone production was decreased when compared to the commercial harvest (Fig. 5).

## Discussion

### Fertility variation and effective number of parents

Each gamete produced by a diploid tree only harbors one allele of each gene, which is chosen at random from the tree’s two copies. Under Mendel’s law of segregation, each of the two alleles in the tree has an equal probability of being included in a gamete. However, the probability is expected to change due to the present fertility variation between female and male parents. The sibling coefficient (Ψ) describes the fertility variation in the population under study as it is derived from the variances of female and male fertility (i.e., coefficient of variation, *CV*_*f*_ and *CV*_*m*_). It does not depend on the genealogical relationship between parents (i.e., related or otherwise: [19]). When all parents, female and male, contribute equally (Ψ = 1), which is proportionate to census number (1/*N*), then the situation of covariance (Fig. 1) is similar to the no covariation as described in Scenario A. The Ψ can also describe the expected increase of inbreeding (i.e.*,* loss of gene diversity) in the seed crops following random mating.

If there is no gene migration (gene flow from outside the orchard), the inbreeding in the following generation will be equal to Ψ/(2 *N*), which is the probability that uniting gametes are identical-by-decent in a random mating population [27]. In a seed orchard of bisexual species, *Pinus tabuliformis*, and over surveyed years, Li et al. [28] reported the presence of significant positive and negative correlations between female and male parents’ contributions. Such correlations should be taken into consideration when the gene diversity of seed crop is estimated because maternal and paternal contribution covariation would mitigate or boost the difference of gametic contribution between gender as shown this study.

The effective number of parents (*N*_*p*_) is expected to be equivalent to the status number (*N*_*s*_) if the population members are non-inbred and unrelated [12, 18, 29, 30]. The *N*_*p*_ is a derivative of effective population sizes to estimate gene diversity in the real population, which considers the variance of contribution (fertility variation) among parents. Gene migration (pollen flow/contamination from outside sources) is expected to increase *N*_*p*_ and gene diversity but decrease orchard crops’ genetic worth [18, 31–35]. It is worth noting that gene migration only affects a portion of the male contribution, which represents half of the seed crops’ parental input.

### Manipulating reproductive output variation through crop management

The reduced effective number of parents and the presence of common parentage (i.e., relatedness among clones) are expected to increase the inbreeding in the resulted seed crops. The parental distortion (i.e., fertility variation) was improved and in turn the effective number of parents was increased. When all crops are pooled across the four-years, indicating that mixing seeds from several years could be beneficial in enhancing gene diversity. While the number of female and male strobili is an indication of gametic contribution among the orchard parents, it should be stated that this assumption can be affected by other factors such as reproductive phenology variation, pollen dispersal distances, pollen viability and competition, self-compatibility, male-female complementarity and/or frequency-dependent male reproductive success as well seed viability and germination [13, 36–38].

Implementation of equal seed-cone harvest caused a substantial loss of seed production (Fig. 4). Thus, a trade-off between seed production and the effective number of parents (gene diversity) should be carefully considered. The fertility from over-represented female parents would be the most concern in the equalizing maternity in seed orchards [1, 39]. The trade-off between gene diversity and seed collection would be more important in the ex-situ gene conservation program of genetic resources [26].

Maternal, paternal, and parental (clonal) contribution can be appropriately estimated by analysis of reproductive output and correlation (covariation) between female and male parents across individuals in a seed orchard. In turn, gametic and genetic contribution of individuals to their seed crops can be calculated [28]. To alter the genetic composition of orchards’ gene pools and improve the genetic worth of their resulting seed crops, intrusive management options can be applied during cone crop development. To effectively manipulate the gene pool, orchard crops’ genetic composition needs to be predicted to assist the decision-making process and the selection of the appropriate management option to implement (e.g., genetic thinning, selective cone harvest: [28, 40]).

## Conclusions

We recommend the use of fertility variation (i.e., *CV* and Ψ), Person’s product-moment correlation (*r*) and effective number of parents (*N*_*p*_) as tools for gauging seed orchard crops’ gene diversity. The effective number of parent (*N*_*p*_) is a characteristic of the seed crops derived from unequally contributing parents. This could be extended to orchard parents in advanced generation seed orchards (or breeding populations) because the *N*_*p*_ does not depend on the relatedness of parents but solely on the fertility variation.

The present study highlighted the presence of some obstacles with female fertility (seed production) and gene diversity loss in the studied 1.5-generation *P. koraiensis* clonal seed orchard, which were mainly associated with large fertility variation, inadequate pollen supply, panmictic disequilibrium, and parental unbalance. Thus, the implementation of seed-cone crops management alternatives such as equal seed-cone harvest among clones and/or supplemental-mass-pollination could be effective options in improving the parental balance and the crop’s genetic worth, and increasing the gene diversity.

## Methods

### Theoretical development of effective number of parents and gene diversity estimation

Parental fertility is defined as the proportional gametic contribution of female and male parents to their progeny [9, 41]. Assuming that female and male strobili production count is a good representative of their gametic contribution [39, 42, 43], this count can then be used to estimate potential gametic contribution and hence parental fertility.

Fertility variation is described by the sibling coefficient (Ψ), which is the probability that two alleles randomly chosen from the gamete gene pool originated from the same parent [19]. Furthermore, the sibling coefficient is connected to the coefficient of variation (*CV*) of female and male reproductive outputs [19, 43]. Female and male parents are defined as those parents contributing female and male gametes, respectively. Thus, the sibling coefficient of parental fertility (Ψ), which is based on zygotes (i.e., seeds), can be further described separately as female (ψ_*f*_) and male (ψ_*m*_) sibling coefficients as:

1.1 $$ {\uppsi}_f=N{\sum}_{i=1}^N{f}_i^2={CV}_f^2+1 $$

1.2 $$ {\uppsi}_m=N{\sum}_{i=1}^N{m}_i^2={CV}_m^2+1 $$

where *N* is the population census number, *f*_*i*_ and *m*_*i*_ are the proportional contributions of female and male of the *i*-th individual, and *CV*_*f*_ and *CV*_*m*_ are the coefficients of variation of female and male reproductive outputs in the population.

The effective number of female (*N*_*p*_^(*f*)^) and male (*N*_*p*_^(*m*)^) parents can be calculated separately from the female (ψ_*f*_) and male (ψ_*m*_) sibling coefficients, and are connected with their respective coefficient of variation (female *CV*_*f*_ and male *CV*_*m*_) [19, 44] as follows:

2.1 $$ {N_p}^{(f)}=\frac{N}{\uppsi_f}=\frac{N}{CV_f^2+1} $$

2.2 $$ {N_p}^{(m)}=\frac{N}{\uppsi_m}=\frac{N}{CV_m^2+1} $$

where *N* is the population census number, ψ_*f*_ and ψ_*m*_ are the female and the male fertility variation (i.e., sibling coefficients), and *CV*_*f*_ and *CV*_*m*_ are the female and male reproductive output’s coefficients of variation in the population under study.

#### Scenario a (dioecious species): no covariation between female and male fertility

When there is no covariation between female and male reproductive outputs, the sibling coefficient (Ψ) is calculated from eqs. (1.1) and (1.2) components as:

3 $$ \Psi =N{\sum}_{i=1}^N{p}_i^2=N{\sum}_{i=1}^N{\left(\frac{f_i+{m}_i}{2}\right)}^2=0.25\left({\uppsi}_f+{\uppsi}_m\right)+0.5 $$

where *N* is the population census number, *p*_*i*_ is the total contribution (fertility) of the *i*-th individual, *f*_*i*_ and *m*_*i*_ are the proportional contributions of the *i*-th individual as female and male parents, and ψ_*f*_ and ψ_*m*_ are the female and male parents’ sibling coefficients, respectively.

The parental effective number of parents (*N*_*p*_) can be calculated from the sibling coefficient (Ψ) (see also formula 2.1 and 2.2). The *N*_*p*_ is equivalent to the status number (*N*_*s*_) when the parents are non-inbred and unrelated [18, 39].

4 $$ {N}_p=\frac{N}{\Psi}=\frac{N}{0.25\left({\uppsi}_f+{\uppsi}_m\right)+1}=\frac{4N}{CVf^2+{CVm}^2+4} $$

where *N* is the population census number, ψ_*f*_ and ψ_*m*_ are the female and male parent’s sibling coefficients, and *CV*_*f*_ and *CV*_*m*_ are the coefficients of variation for female and male reproductive outputs in the population under study, respectively.

#### Scenario B (monoecious or hermaphrodite species): positive or negative correlation between female and male fertility

Under covariation between female and male fertility (i.e., between female and male reproductive outputs), the sibling coefficient (Ψ) can be developed with the Person’s correlation coefficient (*r*) as follows:

5 $$ \Psi =0.25\left({\uppsi}_f+{\uppsi}_m\right)+0.5r\sqrt{\left({\uppsi}_f-1\right)\left({\uppsi}_m-1\right)}+0.5 $$

where ψ_*f*_ and ψ_*m*_ are the female and male parent’s sibling coefficients, and *r* is the Person’s product-moment correlation coefficient between female and male reproductive outputs in the population.

With the covariation (i.e., correlation) between female and male reproductive outputs, the formulae (4) for the parental effective number of parents (*N*_*p*_) can further be developed with the correlation coefficient (*r*) as:

6 $$ {N}_p=\frac{N}{\Psi}=\frac{4N}{\left({\uppsi}_f-1\right)+\left({\uppsi}_m-1\right)+2r\sqrt{\left({\uppsi}_f-1\right)\left({\uppsi}_m-1\right)}+4} $$

where *N* is the population census number, Ψ is the parental sibling coefficient, ψ_*f*_ and ψ_*m*_ are the sibling coefficients of female and male parents, *CV*_*f*_ and *CV*_*m*_ are the female and male reproductive outputs coefficients of variation, and *r* is the Person’s correlation coefficient between female and male reproductive outputs.

Animal breeders and geneticists use the number of fathers (*N*_*f*_) and mothers (*N*_*m*_) to estimate the effective population size as *Ne*^(*v*)^ = 4*N*_*f*_*N*_*m*_ / (*N*_*f*_ + *N*_*m*_) when the sex ratio of a population departs from Fisherian sex ratio (1:1), dealing with dioecies species [14, 17]. In woody plant breeding, however, most gymnosperms are monoecious species so that the correlated fertility between gender should be considered for estimation the effective population size.

In this study, we provided different formula for dioecious species (Scenario A) and monoecious or hermaphrodite species (Scenario B); however, the formulae (4) has the same function when *r* is equal to zero as the formulae (6), so we propose to use the formulae (6) as a general equation of genetic indicator.

### Relative effective number of parents and loss of gene diversity

The relative effective number of parents (*N*_*r*_) is calculated as the relative proportion of the effective number of parents (*N*_*p*_) divided by census number (*N*) and it is a description of the percentage of the real population functioning as the idealized population. It is estimated for female, male and combined parents as:

7 $$ {N}_r\left(\%\right)=\frac{N_p\ }{N}\times 100,\kern0.5em {N_r}^{(f)}\left(\%\right)=\frac{{N_p}^{(f)}\ }{N}\times 100\kern0.5em and\kern0.5em {N_r}^{(m)}\left(\%\right)=\frac{{N_p}^{(m)}\ }{N}\times 100\kern0.5em $$

The loss of gene diversity (*GD*) between generations (from parents to offspring) is estimated following Nei [45], Lacy [46] and Lindgren and Mullin [18] as:

8 $$ GD\ \mathrm{loss}\ \left(\%\right)=\frac{0.5}{N_p}\ \mathrm{x}\ 100 $$

In small populations such as tree seed orchards, the effective population size and the genetic diversity of progeny can be calculated from eqs. 4, 6 and 8. In seed orchards setting, determining the effective population size and the genetic diversity of progeny can be estimated easily using both coefficient of variation (*CV*) and coefficient of correlation (*r*) for parental reproductive outputs (e.g., either strobili, seed-cone or seed production).

### *Pinus koraiensis* seed orchard as a case population

Based on the above-theoretical representation, we estimated the effective number of parents (genetic diversity of the seed crops) and the factors influencing its pattern in the 1.5-generation *Pinus koraiensis* clonal seed orchard. The seed orchard was established by the National Institute of Forest Science, Republic of Korea in 1995 and located in the Gangwon province, South Korea (N37°23′; E127°38′) with 52 clones (total of 713 ramets; average of 37 ramets/clone). Clones/ramets were randomly allocated to the orchard’s grid at 5 × 5 m spacing. The seed orchard is now owned and managed by the National Seed Variety Center of the Korea Forest Service.

Over a consecutive four-year period (2014–2017), the numbers of female and male strobili were assessed for all ramets (100% sampling). The female strobili were individually counted over the entire crown while the numbers of male strobili were estimated by multiplying the average number of strobili per branch by the total number of strobili-bearing branches.

Parental reproductive output balance was assessed using a cumulative gamete contribution curve [9, 38] after sorting the number of female and male strobili produced per clone in descending order and the cumulative contribution percentages were plotted against the proportion of clones.

Equal-cone harvest, collecting equal proportions of cones from each clone, was proposed to mitigate the female fertility variation among clones. The equal-cone harvest among clones was imposed in the seed orchard of *P. koraiensis*, thus the female parents’ fertility variation was negated. It should be noted that equal-cone harvest should be principally given to the most productive clones and thus accepting some loss of cone production is considered.

## Data Availability

The datasets used and/or analyzed during the current study are available from the corresponding author on reasonable request.
